# Immunogenicity and safety of a quadrivalent meningococcal tetanus toxoid-conjugate vaccine (MenACYW-TT) in healthy toddlers: a Phase II randomized study

**DOI:** 10.1080/21645515.2020.1733869

**Published:** 2020-04-01

**Authors:** Timo Vesikari, Ray Borrow, Aino Forsten, Helen Findlow, Mandeep S. Dhingra, Emilia Jordanov

**Affiliations:** a Vaccine Research Center, University of Tampere, Tampere, Finland; b Vaccine Evaluation Unit, Public Health England, Manchester, UK; c Global Clinical Sciences, Sanofi Pasteur, Swiftwater, PA, USA

**Keywords:** Meningococcal, quadrivalent meningococcal conjugate vaccine, menACYW-TT, toddlers

## Abstract

Neisseria meningitidis

can lead to invasive meningococcal disease to which young children are particularly vulnerable. We assessed the immunogenicity and safety of Sanofi Pasteur’s investigational quadrivalent (serogroups A, C, Y, and W) meningococcal tetanus-toxoid conjugate vaccine, MenACYW-TT, as a single dose, in healthy meningococcal vaccine-naïve toddlers versus a licensed conjugate vaccine MCV4-TT (NCT03205358). In this Phase II study conducted in Finland, 188 toddlers aged 12–24 months were randomized 1:1 to MenACYW-TT or MCV4-TT. Serum bactericidal antibody assays using human complement (hSBA) and baby rabbit complement (rSBA) measured antibodies against each serogroup before and 30 days after vaccination. Participants were monitored for immediate adverse events (AEs) and post-vaccination AEs for 30 days. All analyses were descriptive. All 188 participants completed the study. The Day 30 hSBA seroresponses (hSBA titer <8 at baseline and post-vaccination titer ≥8, or ≥8 at baseline and ≥4-fold increase post-vaccination) were comparable between participants receiving MenACYW-TT (96.7–100%), and MCV4-TT (86.0–100.0%) for each serogroup. Most unsolicited AEs were of Grade 1 or Grade 2 intensity. There were no immediate hypersensitivity reactions, and no AEs or serious AEs leading to discontinuation from the study. In this exploratory study, MenACYW-TT vaccine was well tolerated and immunogenic. If confirmed in Phase III, a single dose of the MenACYW-TT vaccine may show promise as an alternative vaccine option for toddlers receiving meningococcal vaccination for the first time.

## Introduction

Invasive meningococcal disease (IMD) is a vaccine-preventable disease caused by *Neisseria meningitidis*, a Gram-negative diplococcus found exclusively in humans. Worldwide, most cases of IMD are caused by six serogroups; A, B, C, W, Y, and X, the distribution of which varies by region.^1–3^ Individuals most at risk of IMD are infants and young children, young adults and those who are immunocompromised.^3–6^

In Europe, the incidence rate of IMD has slowly declined over the period 2000 to 2016, with some studies suggesting that protection from conjugate vaccines have contributed to the decline.^7^ However, the disease remains a serious problem, having an age-standardized rate of 0.64 confirmed cases per 100,000, and a case fatality rate of 10.4% in 2016.^8^ In 2016, 90% of reported cases of IMD in Europe were caused by serogroups B, C, W, and Y, with serogroups B and C the most frequent^8^; however, there has been a rapid expansion of serogroup W across several European countries since an initial variant emerged in the UK in 2013.^9^

In areas of the world where serogroups W and Y are responsible for a significant burden of disease, quadrivalent polysaccharide-conjugate vaccines are available.^2,10^ In these vaccines, the individual serogroup polysaccharide antigens are conjugated to a protein carrier. There are currently three licensed quadrivalent conjugate vaccines against serogroups A, C, W, and Y. Menactra® (MCV4-DT; Sanofi Pasteur, USA), which contains a diphtheria toxoid protein carrier, was licensed in the USA in 2005 for babies aged 9–23 months (administered as two doses three months apart), and for individuals aged 2 to 55 years as a single dose; it is not licensed in Europe.^11,12^ Menveo® (MCV4-CRM; GlaxoSmithKline, Italy), which has *Corynebacterium diphtheriae* CRM_197_ protein as a protein carrier, was licensed in 2010, and is administered as a single dose from age ≥2 years in Europe, with no upper age limit.^13^ Lastly, Nimenrix® (MCV4-TT; Pfizer Europe, Belgium), a polysaccharide-tetanus toxoid conjugate vaccine, was licensed in Europe in 2012, but not in the USA, and is administered as a single dose for infants aged ≥6 weeks with no upper age limit.^14^ Sanofi Pasteur has developed a new quadrivalent conjugate vaccine, MenACYW-TT, which contains a tetanus toxoid protein carrier, intended for use in all individuals aged ≥6 weeks.

This Phase II study was conducted to evaluate the immunogenicity and safety of MenACYW-TT compared with the licensed vaccine MCV4-TT, in healthy toddlers, using both human complement (hSBA) and baby rabbit complement (rSBA) serum bactericidal antibody assays. hSBA titers ≥4 are an accepted surrogate of protection against serogroups A and C.^15^ However, assays using rSBA complement have been used as the basis for licensure of most meningococcal vaccines, with data supporting the acceptance of rSBA titers ≥8 as the correlate of protection against serogroup C.^16^

## Methods

### Study design and participants

This study, MET54, was a Phase II, randomized, active-controlled, open-label study of a single dose of MenACYW-TT, conducted in eight centers in Finland, in meningococcal vaccine-naïve toddlers aged >12 and <24 months. The aim was to evaluate immunogenicity and safety of the vaccine when given alone compared with that of a licensed vaccine MCV4 (EudraCT# 2014-004367-20; NCT03205358). The study was conducted between 31 March 2015 and 19 August 2015.

Participants were aged >12 and <24 months on the day of the first study visit, born at full term of pregnancy (≥37 weeks) or with a birth weight ≥2.5 kg. Exclusion criteria included participation in another clinical trial, any vaccination in the four weeks preceding the study, or planned before the final blood sampling (except for influenza vaccination [≥2 weeks before or after study vaccine]). Other exclusion criteria included previous receipt of any meningococcal vaccine containing serogroups A, B, C, W, or Y, or a history or high risk of meningococcal infection; receipt of immunoglobulins, blood, or blood-derived products in the past three months; known or suspected congenital or acquired immunodeficiency, or receipt of immunosuppressive therapy within the preceding six months; known systemic hypersensitivity or history of a life-threatening reaction to any of the vaccine components; a personal history of Guillain-Barré syndrome or an Arthus-like reaction after vaccination with a tetanus toxoid-containing vaccine.

Parents or legal representatives provided written informed consent for all study participants. The conduct of this study was consistent with standards established by the Declaration of Helsinki and compliant with the International Conference on Harmonization guidelines for good clinical practice as well as with all local and/or national regulations and directives. The study was approved by the National committee of Finland.

Participants were randomized 1:1 via an interactive voice response system to receive one dose of MenACYW-TT or MCV4 control on Day 0. The study had an open-label design as the vaccines had differing appearances; however, the laboratory personnel performing the serology testing were blinded to the group assignment.

MenACYW-TT vaccine was presented in 0.5 mL of saline solution containing 10 μg of each of meningococcal capsular polysaccharides serogroups A, C, Y, and W, and approximately 55 μg of tetanus toxoid protein carrier. The active control was a licensed vaccine MCV4-TT (Nimenrix®, Pfizer Europe, Belgium) and was presented as a powder and solvent for solution for injection, and reconstituted (0.5 mL after reconstitution), to contain 5 μg of each of serogroups A, C, W, and Y, and approximately 44 μg of tetanus toxoid protein carrier.

### Immunogenicity

Blood samples were collected pre-vaccination on Day 0 and on Day 30 (Day 30 to 44 maximum). Oral or injectable antibiotic therapy was not allowed within 72 hours prior to blood withdrawal (blood withdrawals could be postponed but had to still be within the Day 30 to 44 window), and all assays were performed by qualified laboratory personnel.

The primary objective was to evaluate the immunogenicity of the study vaccines against representative test strains for meningococcal serogroups A, C, W, and Y,^17^ as measured by hSBA (Global Clinical Immunology Laboratory; Sanofi Pasteur; Swiftwater, USA) and rSBA (Public Health England; Manchester, UK) assays on Day 0 and Day 30. The same methodology was used for both assays, as per Maslanka *et al*, 1997,^18^ including use of serogroup-specific meningococcal bacteria for all four serogroups. Briefly, two-fold dilutions of test sera were prepared in microtiter plates and meningococcal target strain along with human or baby rabbit complement were added and incubated. Following incubation, 10 μL of the serum/complement/bacteria mixture was added to a blood agar plate, and then incubated. Bacterial colonies present on the blood agar plate were then counted. The bactericidal titer of each sample was expressed as the final reciprocal dilution yielding ≥50% killing compared with the control well.^18^ The lower limit of quantitation (LLOQ) of both assays was a titer of four. Vaccine seroresponse was defined for both hSBA and rSBA as post-vaccination titers ≥8 for participants with pre-vaccination titers <8, or a ≥ 4-fold increase in titers from pre- to post-vaccination for participants with pre-vaccination titers ≥8. Seroprotection for each meningococcal serogroup was defined as post-vaccination hSBA titers of ≥8. An additional definition was also used for defining seroresponse only for rSBA as post-vaccination titers ≥32 for participants with pre-vaccination titers <8, or a ≥ 4-fold increase in titers from pre- to post-vaccination for participants with pre-vaccination titers ≥8.

Both pre- and post-vaccination blood samples were also tested for anti-tetanus antibodies using an enzyme-linked immunosorbent assay (ELISA) and the World Health Organization (WHO) human standard lot TE3 as the reference standard. The LLOQ of the ELISA was 0.01 IU/mL. Geometric mean concentrations (GMCs) were determined for the samples and the proportions of participants achieving levels between ≥0.01 and ≥0.1 IU/mL of antibody concentrations to tetanus toxoid were determined.

### Safety

Participants were kept under observation for 30 minutes after vaccination to ensure their safety. Any unsolicited adverse event (AE) occurring during the first 30 minutes post-vaccination was recorded on the electronic case report form. After this initial time period, parents or legally acceptable representatives were instructed to record specific information about solicited reactions in a diary card, on the day of vaccination and for the seven days immediately after this, in addition to recording any other unsolicited AEs from the day of the vaccination until 30 days after. Unsolicited non-serious AEs were graded on a 3-point intensity scale of ‘Grade 1: No interference with activity’, ‘Grade 2: Some interference with activity’, and ‘Grade 3: Significant, prevents daily activity’. Information on serious AEs (SAEs) were collected and assessed by the Investigator, from inclusion until 30 days after vaccination, through the electronic data capture system. The investigator assessed the causal relationship between the SAE and the investigational product.

### Statistical analyses

This was a descriptive Phase II study to provide safety and immunogenicity data, as such no hypotheses were tested and no formal sample size calculations were performed. The overall planned study cohort (N = 200) provided a probability of approximately 95% of observing any AE with a true incidence of 1.5%; in each group with N = 100, there was a probability of approximately 95% of observing any AE with a true incidence of 3%. No formal statistical hypothesis was tested.

Three analysis sets were used; the full analysis set (FAS), the per-protocol analysis set (PPAS), and the safety analysis set (SafAS). The FAS included all participants who received at least one dose of the study vaccine, and had any valid post-vaccination serology result. The PPAS included all participants from the FAS who adhered to the protocol specific inclusion criteria, and who did not have the deviations from the study protocol; all immunogenicity analyses were performed on the PPAS. The SafAS included participants who had received at least one dose of the study vaccine, and had safety data available.

Categorical data (proportion with hSBA and rSBA antibody titers with a ≥ 4-fold rise from baseline; proportion with hSBA and rSBA titers ≥8 at Day 0 and Day 30, proportion with hSBA and rSBA vaccine seroresponse, and proportion achieving levels between ≥0.01 and ≥0.1 IU/mL of antibody concentrations to tetanus toxoid at Day 0 and 30) are reported as number and proportion of participants with 95% confidence intervals (CIs). Continuous data (titers and concentrations) were reported as log_10_ values using Student’s t distribution with n-1 degree of freedom for mean (standard deviation); anti-Log_10_ transformations were applied to these results to derive geometric mean titers/concentrations (GMTs/GMCs; with 95% CI). For safety data, numbers and proportion (95% CIs) of participants with solicited and unsolicited AEs/SAEs were determined, and the number of unsolicited events was also calculated. Analyses were conducted using SAS version 9.2 or later (SAS Institute Inc.; Cary, NC)

## Results

### Study participants

**Figure 1.**
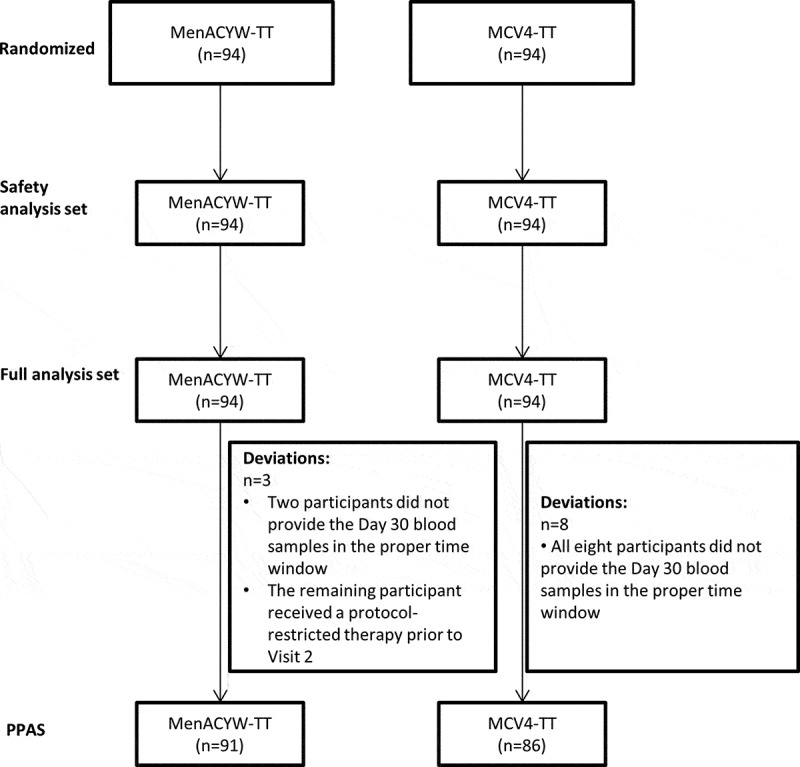
Study groups and protocol deviations.

A total of 188 toddlers were enrolled and randomly allocated to receive either MenACYW-TT (N = 94) or MCV4-TT (N = 94). All participants completed the study. Overall, 177 (94.1%) participants were included in the PPAS (Figure 1). Baseline demographics are summarized in Table 1; participants’ ages were comparable across the groups, however, there were more males than females in the MenACYW-TT group, and more females than males in the MCV4-TT group.

**Table 1.** Baseline demographics by vaccination group (FAS).

	MenACYW-TT (N = 94)	MCV4-TT (N = 94)	All (N = 188)
**Sex, n (%)**			
Male	57 (60.6)	41 (43.6)	98 (52.1)
Female	37 (39.4)	53 (56.4)	90 (47.9)
**Age (years)**			
Mean (SD)	1.44 (0.302)	1.47 (0.314)	1.45 (0.308)

FAS, full analysis set; SD, standard deviation

N, number of participants in assigned group

### Immunogenicity

#### Baseline immune status

At baseline, serogroup-specific meningococcal hSBA GMTs were comparable between vaccine groups (Table 2), and the proportion of participants with meningococcal hSBA titers ≥8 for serogroups A, C, W, and Y ranged from 2.2% to 13.3% in the MenACYW-TT group, and from 0.0% to 14.0% in the MCV4-TT group (Table 3). The proportion of participants with meningococcal rSBA titers ≥8 for serogroup A was 33.0% in the MenACYW-TT group compared with 17.4% in the MCV4-TT group, but was similar between vaccine groups for the other three serogroups (Table 3). Baseline rSBA titers for serogroup A were higher in the MenACYW-TT group compared with the MCV4-TT group (Table 2).

**Table 2.** Geometric mean titers at baseline (Day 0) and Day 30 post-vaccination as assessed by hSBA and rSBA (PPAS).

		hSBA GMT (95% CI)		rSBA GMT (95% CI)	
Serogroup	Time Point	MenACYW-TT (N = 91)	MCV4-TT (N = 86)	MenACYW-TT (N = 91)	MCV4-TT (N = 86)
A	**Day 0**	3.5 (3.1, 3.9)	3.7 (3.3, 4.2)	12.4 (7.1, 21.8)	5.6 (3.4, 9.0)
	**Day 30**	76.8 (63.0, 93.7)	61.5 (45.5, 83.1)	3137.5 (2667.9, 3689.7)	7377.1 (6151.5, 8846.8)
C	**Day 0**	2.4 (2.2, 2.7)	2.5 (2.3, 2.8)	2.2 (1.9, 2.4)	2.3 (2.0, 2.8)
	**Day 30**	492.9 (405.9, 598.5)	28.4 (21.4, 37.5)	2440.1 (2055.4, 2897.0)	418.6 (327.1, 535.5)
W	**Day 0**	2.2 (2.0, 2.5)	2.1 (2.0, 2.1)	2.4 (2.0, 2.9)	2.4 (2.0, 3.0)
	**Day 30**	71.7 (56.3, 91.5)	44.5 (36.6, 54.2)	5306.8 (4318.8, 6520.8)	4333.7 (3520.1, 5335.5)
**Y**	**Day 0**	2.3 (2.0, 2.5)	2.4 (2.1, 2.7)	4.3 (2.9, 6.3)	3.5 (2.4, 4.9)
	**Day 30**	96.6 (75.8, 123.1)	76.4 (61.4, 95.1)	2633.3 (2129.1, 3256.7)	2759.6 (2254.8, 3377.4)

CI, confidence interval; GMT, geometric mean titer; hSBA human complement serum bactericidal antibody assay; PPAS, per-protocol analysis set; rSBA, baby rabbit complement serum bactericidal antibody assay

N: number of participants in assigned group

**Table 3.** Proportions of participants with post-vaccination titers ≥8 against meningococcal serogroups A, C, W, and Y at Day 30, using hSBA and rSBA (PPAS).

	hSBA*, % (95% CI)		rSBA, % (95% CI)	
Serogroup	MenACYW-TT (N = 91)	MCV4-TT (N = 86)	MenACYW-TT (N = 91)	MCV4-TT (N = 86)
A	97.8 (92.3, 99.7)	91.9 (83.9, 96.7)	100 (96.0, 100.0)	100 (95.8, 100.0)
C	100 (96.0, 100.0)	89.5 (81.1, 95.1)	100 (96.0, 100.0)	98.8 (93.7, 100.0)
W	98.9 (94.0, 100.0)	96.5 (90.1, 99.3)	100 (96.0, 100.0)	100 (95.8, 100.0)
Y	98.9 (94.0, 100.0)	100 (95.8, 100.0)	100 (96.0, 100.0)	100 (95.8, 100.0)

*Seroprotection was defined as post-vaccination hSBA titers of ≥8

CI, confidence interval; hSBA human complement serum bactericidal antibody assay; PPAS, per-protocol analysis set; rSBA, baby rabbit complement serum bactericidal antibody assay

#### Vaccine response

**Figure 2.**
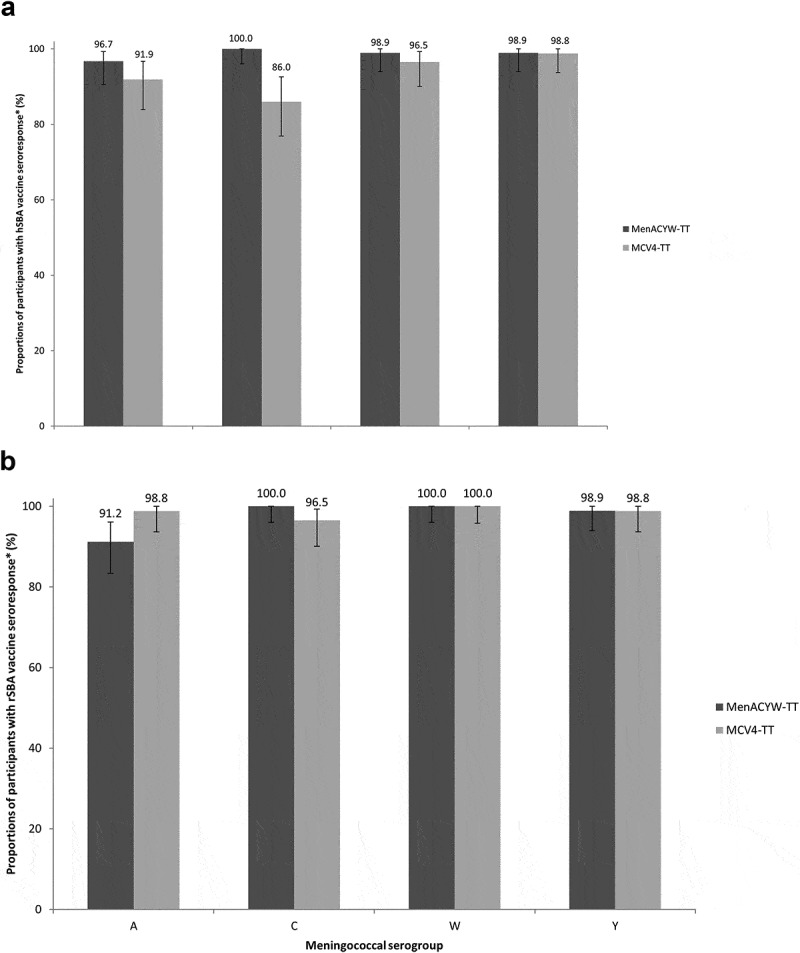
Proportion of participants with vaccine seroresponse at Day 30, against meningococcal serogroups A, C, W, and Y (PPAS) as assessed by (A) hSBA* and (B) rSBA†.

By Day 30, serogroup-specific meningococcal hSBA GMTs had increased similarly in each group (Table 2). The proportion of participants with seroprotection at Day 30 was >90% for each serogroup in both vaccine groups (Table 3). For serogroup C, 100% of participants had hSBA titers ≥8 in the MenACYW-TT group compared with 89.5% in the MCV4-TT group (Table 3). hSBA vaccine seroresponse was seen at Day 30 for most participants in both vaccine groups, across all four serogroups (Figure 2A). The proportion of participants with a hSBA vaccine seroresponse was similar in both groups for serogroups A, W, and Y, but was higher for serogroup C in the MenACYW-TT group (100%) than in the MCV4-TT group (86.0%) (Figure 2A). Results were similar when data for the FAS were considered (Supplementary Table 1; Supplementary Figure 1).

Results for meningococcal rSBA GMTs mirrored the hSBA GMTs (Table 2). Thirty days after vaccination, the proportion of participants with rSBA titers ≥8 increased from baseline for each serogroup in both groups, and was similar between both vaccine groups (Table 3). Most participants in each group demonstrated an rSBA vaccine seroresponse at Day 30. The proportion of participants with rSBA vaccine seroresponse at Day 30 for serogroup A was lower in the MenACYW-TT group than the MCV4-TT group (Figure 2B), though the two groups differed at baseline. The proportion of participants with rSBA vaccine seroresponse, was similar between the two groups for serogroups C, W, and Y (Figure 2B). The rSBA seroresponse was lower in the MenACYW-TT group for serogroup A, however, the baseline titers for serogroup A were higher in the MenACYW-TT group compared with the MCV4-TT group. Results were similar when data for the FAS were considered (Supplementary Table 1; Supplementary Figure 1).

GMCs of anti-tetanus antibodies measured by ELISA were similar in the MenACYW-TT (1.6 IU/mL [95% CI 1.3, 2.1]) and MCV4-TT (1.4 IU/mL [95% CI 1.2, 1.8]) groups at baseline. On Day 30 post-vaccination, GMCs of anti-tetanus antibodies measured by ELISA had increased, but remained similar between the MenACYW-TT and MCV4-TT groups (11.5 IU/mL [95% CI 9.6, 13.8] and 9.5 IU/mL [95% CI 7.9, 11.4], respectively). At both baseline and Day 30 post-vaccination, all toddlers had seroprotective concentrations (≥0.1 IU/mL) in both vaccine groups.

### Safety

#### Immediate adverse events

There were no immediate unsolicited AEs or reactions after vaccination in either vaccine group (Table 4).

**Table 4.** Summary of safety data from Day 0 to Day 30 post-vaccination with MenACYW-TT or MCV4-TT (SafAS).

	MenACYW-TT, % (95% CI) (N = 94)	MCV4-TT, % (95% CI) (N = 94)
**Immediate**		
Unsolicited systemic AE	0 (0.0, 3.8)	0 (0.0, 3.8)
Unsolicted AR	0 (0.0, 3.8)	0 (0.0, 3.8)
Solicited reaction^*†^	79.8 (70.2, 87.4)	83.0 (73.8, 89.9)
Injection site reaction	48.9 (38.5, 59.5)	53.2 (42.6, 63.6)
Grade 3	3.2 (0.7, 9.0)	4.3 (1.2, 10.5)
Tenderness^‡^	29.8 (20.8, 40.1)	33.0 (23.6, 43.4)
Erythema^‡^	30.9 (21.7, 41.2)	35.1 (25.5, 45.6)
Swelling^‡^	14.9 (8.4, 23.7)	18.1 (10.9, 27.4)
Systemic reaction	61.7 (51.1, 71.5)	69.1 (58.8, 78.3)
Grade 3	3.2 (0.7, 9.0)	3.2 (0.7, 9.0)
Fever^‡^	7.4 (3.0, 14.7)	4.4 (1.2, 10.9)
Vomiting^‡^	4.3 (1.2; 10.5)	5.3 (1.7, 12.0)
Abnormal crying^‡^	33.0 (23.6; 43.4)	39.4 (29.4, 50.0)
Drowsiness^‡^	34.0 (24.6, 44.5)	27.7 (18.9, 37.8)
Loss of appetite^‡^	23.4 (15.3, 33.3)	36.2 (26.5, 46.7)
Irritability^‡^	52.1 (41.6, 62.5)	56.4 (45.8, 66.6)
**Unsolicited AE^†^**	58.5 (47.9, 68.6)	60.6 (50.0, 70.6)
Grade 3 unsolicited non-serious AE	3.2 (0.7, 9.0)	3.2 (0.7, 9.0)
**Unsolicited AR^†^**	5.3 (1.7, 12.0)	6.4 (2.4, 13.4)
Grade 3 unsolicited non-serious AR	0.0 (0.0, 3.8)	0.0 (0.0, 3.8)
**SAE**^¶^	1.1 (0.0, 5.8)^a^	0.0 (0.0, 3.8)
**Death**	0.0 (0.0, 3.8)	0.0 (0.0, 3.8)

*All solicited reactions were considered study vaccine related; ^†^ No AE or AR led to study discontinuation; ^‡^ Any grade; ^¶^ No SAE was considered related to study vaccine; no SAE lead to study discontinuation

AE, adverse event; AR, adverse reaction; CI, confidence interval, SAE, serious adverse event; SafAS, safety analysis set

#### Solicited reactions

Solicited injection site and systemic reactions reported between Day 0 and 7 are summarized in Table 4. In both vaccine groups the majority of injection site reactions were Grade 1, all started between Day 0 and Day 3 post-vaccination, and most lasted one to three days. Most solicited systemic reactions were Grade 1 or Grade 2 in intensity, started between Day 0 and Day 3 post-vaccination and lasted one to three days.

#### Unsolicited adverse events and reactions

Unsolicited ARs and AEs reported between Days 0 and 30 are summarized in Table 4. All unsolicited AEs and ARs were non-serious, and most were Grade 1 or Grade 2 in intensity. No unsolicited non-serious injection site ARs were reported following administration of MenACYW-TT, but these events were reported for three (3.2%) toddlers who received MCV4-TT (induration, n = 2 and urticaria, n = 1). At least one unsolicited non-serious systemic AR was reported for 5.3% (5/94) of toddlers who received MenACYW-TT and 4.3% (4/94) of whom received MCV4-TT. The most frequently reported unsolicited AR was diarrhea, reported by 4.3% (4/94) of participants in the MenACYW-TT group and 2.1% (2/94) in the MCV4-TT group, according to system organ class. One toddler who received MenACYW-TT experienced two SAEs (accidental injuries), neither of which was considered related to the vaccine.

There were no AEs, SAEs or ARs that led to discontinuation from the study, and there were no reported deaths.

## Discussion

Here we report that in healthy, meningococcal-vaccine naïve toddlers aged >12 and <24 months in Finland the immunogenicity and safety of a new meningococcal conjugate vaccine, MenACYW-TT, was comparable with that of a licensed meningococcal conjugate vaccine. Seroresponse rates and antibody titers following vaccination were high in both vaccine groups, although there was some variation between the vaccines by serogroup and assay used; toddlers receiving MenACYW-TT had higher hSBA seroresponses to serogroup C post-vaccination, and those receiving MCV4-TT had higher rSBA seroresponses and GMTs to serogroup A, although baseline GMTs for serogroup A also differed between the vaccine groups.

The difference in hSBA assay and rSBA assay results for serogroup A was consistent with previously published literature demonstrating the lack of a strong correlation between the two assays.^19,20^ Complement-mediated bactericidal activity assays measure the ability of serum to kill meningococci in the presence of complement, and this key difference between the two sources of complement is thought to account for the lack of correlation between the assays.^21^ Both SBA assays and GMTs were used due to differing preferences globally for licensing data; the first MCV4 was licensed by the US Food and Drug Administration in 2005 based on rSBA assay data,^16,22^ however, in Europe, MCV4-CRM was licensed in 2009 on the basis of hSBA assay data, with some rSBA assay data in the assessment report,^16,23^ whilst MCV4-TT in 2012 relied primarily on rSBA assay results, with some hSBA assay data.^16,24^

All participants in both groups had seroprotective levels of tetanus toxoid antibody concentrations at baseline. Anti-tetanus antibody GMCs increased in both vaccine groups, albeit with numerically higher responses in the MenACYW-TT group, consistent with a higher tetanus toxoid concentration in this vaccine. This is consistent with results obtained for other conjugate vaccines that use tetanus toxoid as a carrier protein; in particular the serogroup A meningococcal conjugate vaccine, PsA-TT, generated robust tetanus serologic responses in individuals 1 to 29-year-olds,^25^ similar to those expected after a booster dose of tetanus toxoid.^25^ This study showed improved community immunity to tetanus due to PsA-TT campaigns.^25^ However, these findings do not bypass the need for tetanus boosters, rather they indicate there is no adverse impact on tetanus immunogenicity when the tetanus toxoid protein is used as a carrier. Similar phenomenon is observed for diphtheria antibody levels when other meningococcal conjugate vaccines which contain either Diphtheria toxoid or CRM_197_ protein as the conjugate are administered.^26,27^

The study was planned as a descriptive Phase II study to provide safety and immunogenicity data; hence no formal sample size calculations linked to a specific statistical hypothesis were performed. After the study was started, limitations included recruitment in the allotted time period becoming a challenge and resulted in ending enrollment at 188 participants, prior to randomization of the planned 200 participants. Neither this nor open-label design likely had any effect on the observed result. Other limitations included the imbalanced gender ratio, which is known to have an effect on immunogenicity, as males have been found to be more susceptible to IMD than females.^28^

This Phase II study shows that the MenACYW-TT vaccine induced an immune response in toddlers comparable to MCV4-TT across all four meningococcal serogroups. If the results of this exploratory study are confirmed in Phase III, a single dose of MenACYW-TT can be considered as an alternative vaccination option for toddlers receiving meningococcal vaccination for the first time.

## Supplementary Material

Supplemental Material

## Data Sharing

Qualified researchers may request access to patient level data and related study documents including the clinical study report, study protocol with any amendments, blank case report form, statistical analysis plan, and dataset specifications. Patient level data will be anonymized and study documents will be redacted to protect the privacy of trial participants. Further details on Sanofi’s data sharing criteria, eligible studies, and process for requesting access can be found at: https://www.clinicalstudydatarequest.com.
