# Supplementary material for: Immunogenicity and safety of a quadrivalent meningococcal tetanus toxoid-conjugate vaccine (MenACYW-TT) in healthy toddlers: a Phase II randomized study
Source: Hum Vaccin Immunother. 2020 Apr 1;16(6):1306–12. doi: 10.1080/21645515.2020.1733869 (PMC7538019; doi:10.1080/21645515.2020.1733869)
Supplement: Supplemental Material [file KHVI_A_1733869_SM3843.docx]

### Supplementary Table 1. Proportions of participants with post-vaccination titers ≥8 against meningococcal serogroups A, C, W, and Y at Day 30, as assessed by hSBA and rSBA (FAS)

|  | **hSBA^*^, % (95% CI)** | | **rSBA, % (95% CI)** | |
| --- | --- | --- | --- | --- |
| **Titers ≥8, % (95% CI)** | **MenACYW-TT**  **(N=94)** | **MCV4-TT**  **(N=94)** | **MenACYW-TT**  **(N=94)** | **MCV4-TT**  **(N=94)** |
| **A** | 97.9 (92.5, 99.7) | 91.5 (83.9, 96.3) | 100.0 (96.2, 100) | 100.0 (96.2, 100) |
| **C** | 100.0 (96.2, 100.0) | 88.3 (80.0, 94.0) | 100.0 (96.2, 100.0) | 98.9 (94.2, 100.0) |
| **W** | 98.9 (94.2, 100.0) | 96.8 (91.0, 99.3) | 100.0 (96.2, 100.0) | 98.9 (94.2, 100.0) |
| **Y** | 98.9 (94.2, 100.0) | 100.0 (96.2, 100.0) | 100.0 (96.2, 100.0) | 100.0 (96.2, 100.0) |

^*^Seroprotection was defined as post-vaccination hSBA titers of ≥8

CI, confidence interval; FAS, full analysis set; hSBA human complement serum bactericidal antibody assay; rSBA, baby rabbit complement serum bactericidal antibody assay

**Supplementary Figure 1.** Proportion of participants with vaccine seroresponse at Day 30, against meningococcal serogroups A, C, W, and Y as assessed by (A) hSBA and (B) rSBA (FAS)

**A**

**B**

^*^hSBA seroresponse was defined as titer <8 at baseline with post-vaccination titer ≥8 or titer is ≥8 at baseline with a ≥4-fold increase at post-vaccination FAS, full analysis set; †rSBA seroresponse was defined as <8 at baseline with post-vaccination titer ≥32 or titer is ≥8 at baseline with a ≥4-fold increase at post-vaccination

FAS, full analysis set; hSBA human complement serum bactericidal antibody assay; rSBA, baby rabbit complement serum bactericidal antibody assay
